# New terminology of medication adherence enabling and supporting activities: ENABLE terminology

**DOI:** 10.3389/fphar.2023.1254291

**Published:** 2023-10-13

**Authors:** Przemyslaw Kardas, Emma Aarnio, Tamas Agh, Job F. M. van Boven, Alexandra Lelia Dima, Cristina Mihaela Ghiciuc, Fatjona Kamberi, Guenka Ivanova Petrova, Urska Nabergoj Makovec, Indrė Trečiokienė

**Affiliations:** ^1^ Department of Family Medicine, Medical University of Lodz, Lodz, Poland; ^2^ School of Pharmacy, Faculty of Health Sciences, University of Eastern Finland, Kuopio, Finland; ^3^ Syreon Research Institute, Budapest, Hungary; ^4^ Department of Clinical Pharmacy and Pharmacology, University Medical Center Groningen, University of Groningen, Groningen, Netherlands; ^5^ Health Technology Assessment in Primary Care and Mental Health (PRISMA), Institut de Recerca Sant Joan de Déu (IRSJD), Esplugues de Llobregat, Spain; ^6^ Pharmacology, Clinical Pharmacology and Algeziology, Faculty of Medicine, Grigore T. Popa University of Medicine and Pharmacy of Iasi, Iași, Romania; ^7^ Scientific Research Centre for Public Health, Faculty of Health, University of Vlore “Ismail Qemali”, Vlore, Albania; ^8^ Medical University Sofia, Sofia, Bulgaria; ^9^ Faculty of Pharmacy, University of Ljubljana, Ljubljana, Slovenia; ^10^ Pharmacy and Pharmacology Center, Institute of Biomedical Sciences, Faculty of Medicine, Vilnius University, Vilnius, Lithuania

**Keywords:** medication adherence, non-adherence, interventions, technology, drugs, terminology, taxonomy, health policy

## Abstract

**Introduction:** Medication non-adherence negatively affects the effectiveness of evidence-based therapies and sustainability of healthcare systems. Lack of agreed terminology of medication adherence enabling and supporting activities leads to underuse of the available tools. The ENABLE COST Action was aimed at proposing a new terminology for these activities in order to help both scientific research and its clinical application.

**Methods:** Initial discussions within the ENABLE Working Groups allowed for the conceptualization of four interlinked terms related to adherence, i.e., “medication adherence technology”, “medication adherence enhancing intervention”, “best practice” and “reimbursement”. The iterative process of internal discussion was structured around two dedicated international workshops. Moreover, extensive stakeholder consultations have been organised, including an interactive online survey used to assess the level of agreement with, and the clarity of relevant terms and definitions proposed.

**Results:** Detailed analysis of the results of this process allowed for fine-tuning of the items, and finally, for proposing the final set of definitions. Across all the three phases of this process, the definitions were substantially modified to better reflect the concepts, simplify the language, and assure completeness and cohesiveness of terminology. Feedback obtained from the stakeholders helped this process and confirmed that the final terms and definitions were well received by the experts active in the field of medication adherence.

**Discussion:** Covering the gap in the existing terminology, this work proposes a cohesive set of terms and definitions applicable to medication adherence enabling and supporting activities. Promoting evidence-based approach to this field, this terminology may help research, clinical practice and policy.

## Introduction

Medication adherence is a basic precondition for full effectiveness of evidence-based therapies, and therefore, a key enabler of optimal health outcomes. Indeed, non-adherence leads to profound health and economic consequences at both individual and societal levels. Perhaps, the most important one is reduced treatment effectiveness. As expressed in the well-known quote of Dr. C. Everett Koop, a former Surgeon General of the United States, “Drugs do not work in patients who do not take them”. Unfortunately, the more potent is the medication, the greater are losses suffered by the patients deviating from advised therapy, including the fatal consequence of fully preventable premature death (Neto et al., 2021; Mafruhah et al., 2023). Moreover, non-adherence increases the risk of complications associated with the underlying conditions. It also leads to impaired patient quality of life, due to both distressful symptoms as well as limitations in daily activities. Consequently, non-adherence leads to increased healthcare costs due to the need for additional appointments, tests, treatments, hospitalizations, institutionalizations, *etc.* According to a dedicated report by the Organisation for Economic Co-operation and Development (OECD), non-adherence contributes to nearly 200,000 premature deaths in Europe, and generates up to EUR 125 billion annual costs in excess healthcare services (Khan and Socha-Dietrich, 2018). Apart from that, it generates tremendous indirect costs resulting from mortality, absenteeism and the reduced productivity of employees.

Thus, addressing non-adherence is crucial not only for improving patient outcomes and quality of life but also for reducing healthcare costs, promoting better use of pharmaceuticals and fostering a sustainable healthcare system. For these reasons, the problem of non-adherence has been extensively studied for the last 50 years. Unfortunately, despite the number of scientific papers published on that subject grossly exceeding 100,000, we are still far from finding an ultimate solution (Kardas et al., 2023). In real-life settings, non-adherence to medication is still highly prevalent. The seminal World Health Organization (WHO) report, published 20 years ago, estimated that only 50% of patients with chronic diseases adhered to their medication regimens (World Health Organization, 2003). Regrettably, current statistics of non-adherence are very similar (Foley et al., 2021). High prevalence of non-adherence is still the case across a number of conditions, even in life-threatening ones, such as HIV/AIDS (Konstantinou et al., 2020).

What is worse, despite the fact that there are a lot potentially effective interventions available, they are implemented very rarely (Kardas et al., 2022). This is, at least partly, due to lack of relevant terminology, which hinders knowledge transfer between research and practice and results in underuse of available tools. A compelling example of this scenario is evident in the absence of a specific definition for adherence-targeting activities, even within well-cited systematic reviews (Nieuwlaat et al., 2014; Mbuagbaw et al., 2015; Mistry et al., 2015; Morrissey et al., 2016). A recent study, focused on identifying reimbursed medication adherence enhancing interventions across European countries, encountered a significant obstacle due to the absence of a standardized definition for such interventions. This lack of clarity not only posed challenges in identifying reimbursed adherence interventions, but also complicated determinations about which programs should be categorized as adherence-focused (Ágh et al., 2022). Undoubtedly, standardizing of what is called the best practice, interventions, and technology could provide a solution to this problem.

For more than a decade, the ABC taxonomy has stood for a consensus terminology which provides basic definitions to medication adherence area (Vrijens et al., 2012). The ABC taxonomy has been further applied in the EMERGE guidelines which set the standards for reporting scientific studies on medication adherence (De Geest et al., 2018). Unfortunately, no agreement has yet been reached on the terminology applicable to activities aimed at improving medication adherence. This is an important barrier towards fair benchmarking of current interventions, showcasing identified technologies, and stimulating their wider implementation and reimbursement in different healthcare systems. Consequently, the adoption of more effective ways of supporting medication adherence is halted or slowed down by the lack of relevant terminology. Therefore, the alignment and consensus on these terms is of key importance for the advancement of this field.

The European Network to Advance Best practices and technoLogy on medication adherencE (ENABLE) is a multinational research collaboration supported by COST Action (CA19132). It is aimed at facilitating a more rapid and efficient transformation of healthcare systems towards better adherence support. The main goal of ENABLE is to facilitate the adoption and use of medication adherence best practices and technologies by health systems across Europe. This goal is currently being pursued by fostering knowledge on medication adherence, raising awareness of adherence enhancing solutions, accelerating clinical application of novel technologies, and working collaboratively towards economically viable policy, and implementation of adherence enhancing technology across healthcare systems. (van Boven et al., 2021).

Owing to the unprecedented engagement of the stakeholders, ENABLE is well placed to tackle the problem of the lack of consensus on terminology for medication adherence supporting activities. As a unique platform for collaboration and networking of experts interested in medication adherence, currently ENABLE comprises over 200 members from 40 countries (39 European ones and Israel), of which a majority are researchers active in this field. Moreover, ENABLE engages a range of other stakeholders, including healthcare professionals, such as physicians, pharmacists, psychologists, and nurses, patient representatives and advocacy partners, regulatory bodies such as registration authorities, payers, health insurance policymakers, as well as healthcare equipment manufacturers and IT companies.

Activities of ENABLE are organised around 4 Working Groups (WGs), focused on interlinked areas, namely,: mapping best practices available in European countries (WG1 Current Practices and Unmet Needs), identifying and showcasing adherence technologies (WG2 Adherence Enhancing Technologies), identifying suitable reimbursement strategies for implementation of medication adherence interventions in healthcare systems (WG3 Sustainable Implementation of Adherence Enhancing Technologies), and communication and dissemination activities (WG4 Communication and Dissemination).

Thus, the structure of the action provides a practical and goal-oriented framework to agree on consensus terminology through extensive stakeholder consultations. The context of the ENABLE network offers several opportunities, i.e., representation of numerous countries and multiple expertise backgrounds, networking funding instruments (workshops and networking activities), as well as constraints of time and resources.

In this paper, we report on the process of developing a cohesive set of relevant terminology regarding medication adherence supporting activities based on stakeholder consultation, with a view to creating a conceptual framework to coordinate ENABLE activities, and more broadly, proposing this framework for further stakeholder input, and ultimately for guiding research and practice on medication adherence.

## Methods

**FIGURE 1:**
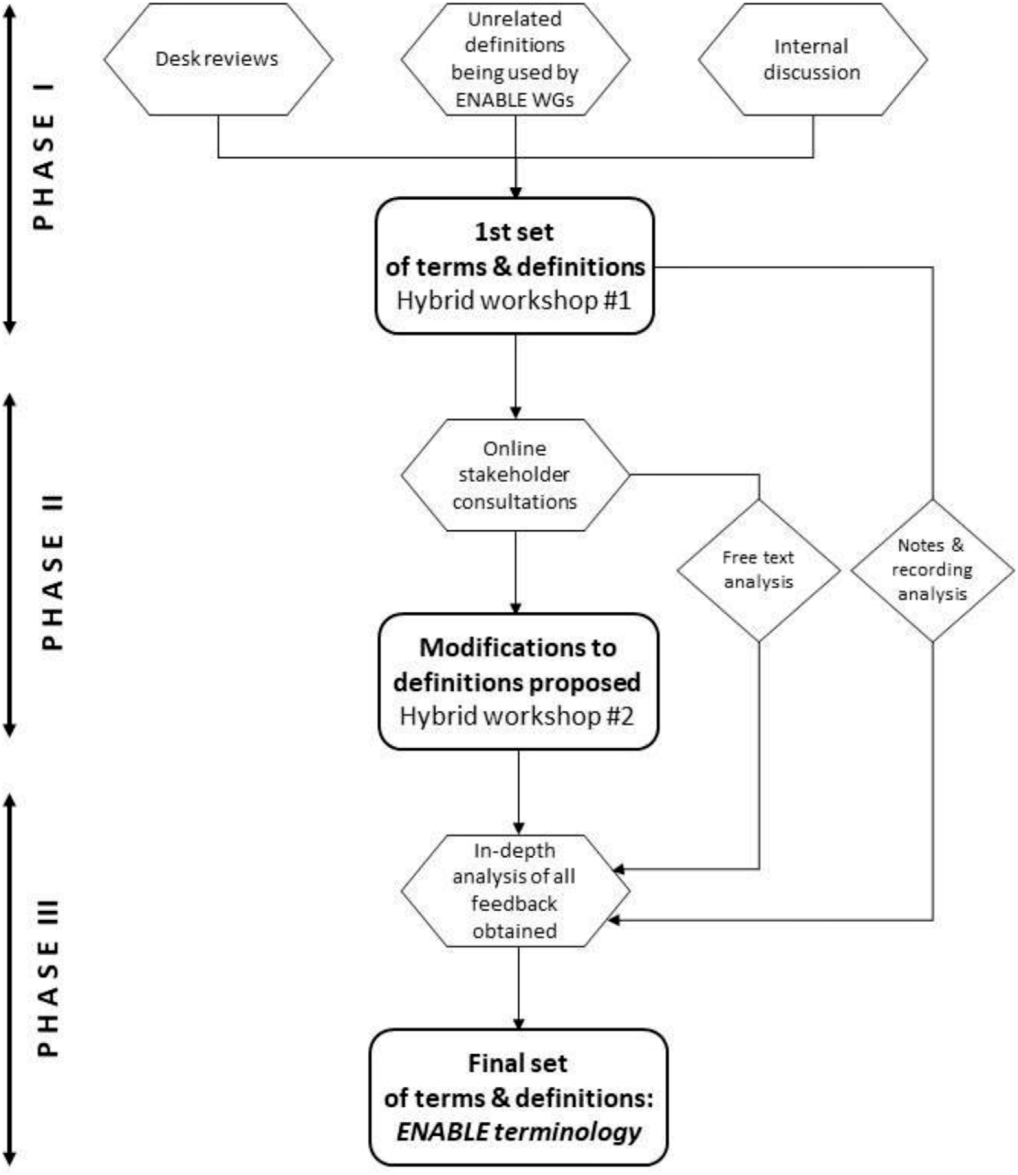
Flow chart of the methodology of the terminology agreeing process.

Terms and definitions constituting terminology of medication adherence enabling and supporting activities were drafted. They were fine-tuned and agreed according to an iterative process illustrated in Figure 1. Details of the process are described below.

### Phase I: problem description and first set of definitions

Internal discussion on the forum of four WGs of ENABLE was a starting point to agree on the terms and definitions of target terminology of adherence enabling and supporting activities through an iterative process. Since October 2020, each WG had developed various activities following their specific objectives and planned outputs, for which they have adopted and defined/operationalized terms relevant for their focus. These definitions, however, were neither consulted across the WGs, nor constituted a cohesive set of terminology. Therefore, during the first year of the Action, it become apparent that, in order to maximise the impact of each output, coordinated action is necessary to align terminology and use it to describe the overall process of generating and implementing medication adherence research into routine practice sustainably and at scale.

Therefore, in early 2022, work on aligning terminology was initiated. Meetings between WGs coordinators allowed to formulate the problem of lack of adherence enabling and supporting activities terminology and set the goal of aligning definitions. This process followed up on the work related to the development of the ENABLE repository framework (Nabergoj Makovec et al., 2022), which was based on the principles of good practice in ontology relevant to development of behaviour change interventions as described by Wright et al. (Wright et al., 2020). That strategy involved defining the scope and key entities of the framework, an iterative process of literature annotation, discussion and revision, expert stakeholder review, disseminating and maintaining the framework.

Discussion within and across the groups was facilitated by desk reviews conducted by each WG when searching the existing terminology items and relevant definitions among the documents known to the team members. These reviews were informed by the standards accepted in the field of medication adherence research, in particular the ABC terminology and taxonomy (Vrijens et al., 2012), and the EMERGE guidelines (De Geest et al., 2018).

These steps allowed for drafting the first set of terms and definitions of adherence enabling activities. In order to facilitate a critical discussion about these results by all relevant stakeholders, a dedicated ENABLE workshop #1 was organised in a hybrid form in Malaga, Spain on 3 May 2022. Along with ENABLE, other professional associations were invited to join the meeting either onsite or online, e.g., the International Society for Medication Adherence (ESPACOMP), the International Society for Pharmacoeconomics and Outcomes Research (ISPOR), and particularly, ISPOR’s Medication Adherence and Persistence Special Interest Group, and European Drug Utilisation Group (EuroDURG) of International Society of Pharmacoepidemiology. The program of the workshop allowed for detailed presentation of each piece of proposed terminology in the context of the activities taken by relevant WGs.

### Phase II: public consultations

Along with *ad hoc* discussion at the forum of the participants of the ENABLE hybrid workshop #1, which was recorded for future reference, a dedicated exercise was organised during this workshop to collect stakeholders’ feedback on presented terms and definitions. Namely, an anonymous interactive survey was conducted among workshop participants, based on provided instructions. The survey, powered by the eDelphi software (eDelphi.org), was made available online. Individual questions concerned basic participants’ characteristics, as well as their level of agreement with, and the clarity of relevant terms and definitions presented. Whenever relevant, individual items were assessed with visual scales, allowing to select a location on a 9-point two dimensional graph representing levels of agreement and clarity. The respondents could also share their free text comments on every item in the survey. Stakeholders who were not able to participate in the consultations in real time could fill in the survey later. To further encourage online participation, additional advertising efforts were directed towards engagement of the stakeholders’ community.

Results of the survey were analysed with descriptive statistics, similarly to those described in more detail elsewhere (Nabergoj Makovec et al., 2022). The respondents’ socio-demographic characteristics were summarized in terms of level of education, profession, primary field of work, years of experience, age, gender, country and participation in ENABLE. The level of agreement on the terms and definitions proposed was described for each item for both relevance and clarity, using the interpercentile range adjusted for symmetry (IPRAS), and the disagreement index (DI), *i.e.*, the ratio between the interpercentile range (IPR) and IPRAS. We considered DI > 1 (*i.e.*, IPR > IPRAS) as indicating disagreement. The median values of relevance and clarity and the DI was used to define different levels of agreement and steer decisions on modifying terms and definitions, as described in (Nabergoj Makovec et al., 2022). In order to analyse whether there were any patterns or differences in the level of consensus reached during voting according to respondents’ characteristics, relevant variables with acceptable distributions were examined using Wilcoxon rank sum tests with Bonferroni correction for multiple testing.

All this allowed for the critical analysis of the definitions provisionally agreed on in Phase I. This analysis was informed by the feedback obtained from both internal (ENABLE community) and external resources. Results of the analysis were presented at the next round of public stakeholder consultations, in a dedicated ENABLE workshop #2, organised in a hybrid form in Oslo, Norway, on 25 June 2022. During the meeting, the participants were updated on the process and provisional outcomes of the drafted set of terminology definitions. The feedback provided by the participants of online stakeholder consultations was overviewed, and their free text comments were summarised. This was followed by open discussion on every item of the proposed terminology, which was voice-recorded. Online chat with remote participants was also recorded for further analysis.

### Phase III: in-depth analysis of stakeholder feedback and final fine-tuning of definitions

The final round of fine-tuning of the terms and definitions included an in-depth analysis of all the sources of feedback provided by the stakeholders in Phase I and II, namely:

1) Verbatim transcript of recording of discussion and hand notes taken at Hybrid workshop #1
2) Verbatim transcript of recording of discussion and chat notes taken at Hybrid workshop #2
3) Free text comments collected during online stakeholder consultations

All these resources were subject to a stepwise qualitative content analysis. In the first step, individual comment items were extracted from the verbatim transcript and transferred to a spreadsheet. Then, the items were ascribed one or more corresponding terminology elements, to allow for their assessment according to the owner. The original spreadsheet was divided into parts belonging to each Working Group, which clustered the comments of similar thematic content, and ascribed each cluster one of the three values: high importance (“very useful”), medium importance (“to be considered”), and low importance (“not important at all”). Results of this process were provided for cross-check and approval by another Working Group (e.g., clusters created by WG1 were approved by WG2, *etc.*). Only high importance items were to be considered in the final round of terminology fine-tuning.

In the final step, approved clustering results were discussed at the forum of cross-WG terminology working panel, which allowed to agree the final wording of the terminology definitions. Additionally, consensus on the definition of best practice was reached via an online tool.

## Results

Initial definitions adopted by various WGs of ENABLE (“Phase 0 definitions”, see Table 1) and used as a starting point for this process, are described below.

• Working on a repository of Medication Adherence Technologies (MATechs), WG 2 adopted a definition of MATech (Nabergoj Makovec et al., 2022) The initial definition of MATech was informed by i) the WHO definition of health technologies (World Health Organization WHO, 2007), ii) the ABC definition of medication adherence (Vrijens et al., 2012) and iii) the WHO definition of adherence to long-term therapies (World Health Organization, 2003)
• For the purpose of their search of reimbursed Medication Adherence Enhancing Intervention (MAEIs) across Europe, WG 3 adopted definitions of MAEI as well as ‘Reimbursement’ applicable to medication adherence enabling and supporting activities (Kardas et al., 2022)
• The discussion over the definition of ‘Best Practices’ applicable to medication adherence enabling and supporting activities adopted by WG 1 was guided by the definition of best practice in healthcare proposed by the European Commission (Perleth et al., 2001).

**TABLE 1:** Terminology of medication adherence enabling and supporting activities–across the phases of the fine-tuning process.

Phase	Item	Definition
PHASE 0	MATech	MATech are devices, procedures or systems developed based on evidence to support patients to take their medication as agreed with the healthcare providers (to initiate, implement and persist with medical regimen)
	MAEIs	MAEIs are broadly defined as any formalised activities taking place within, or in association with the healthcare system, that in any way could positively affect medication adherence at the individual patient level
	REIMBURSEMENT	Reimbursed MAEIs are those MAEIs which are subject to reimbursement by various organizations, such as public healthcare systems, governments, public or private insurance options, pharmaceutical companies, patient organizations, or others. However, interventions paid only through out-of-pocket by individual patients are not regarded as ‘reimbursed MAEIs’
PHASE I	MATech	Medication Adherence Technologies (MATech) are evidence-based health technologies (i.e., devices, techniques, procedures/services, or systems) used in management of medication adherence by diverse stakeholders (i.e., patients, caregivers, healthcare professionals, *etc.*)
	MAEIs	MAEIs are any formalised activities taking place within, or in association with the healthcare system, that in any way could positively affect medication adherence at the individual patient level
	REIMBURSEMENT	Reimbursement relates to public or private insurers’ payment to providers for covering the costs of delivering MATechs and/or MAEIs
**Phase III**	**MATech**	**Medication Adherence Technologies (MATech) are evidence-based health technologies used in the management of medication adherence by different stakeholders**
	**MAEIs**	**MAEIs are any structured activities taking place within, or in association with the healthcare system that have evidence on their positive effect on medication adherence at the individual patient level**
	**REIMBURSEMENT**	**Reimbursement refers to payments made to providers or patients by relevant stakeholders to cover, partly or entirely, the costs of providing MATechs and/or MAEIs**
	**BEST PRACTICE**	**Best practice in medication adherence is evidence-based practice enhancing medication adherence**

Compared to these initial definitions applied by the ENABLE Working Group prior to the terminology design process, the definitions were modified significantly in the iterative process of fine-tuning in Phases I, II and III, as described below.

### MATech phase I definition

For the Phase I consultation on definitions during the Hybrid workshop #1, the MATech definition v2 was used. This version was the upgraded version of ‘Phase 0’ v1 definition, being informed by the results of the Delphi survey conducted for the benefit of the planned repository of MATechs.

**Medication adherence technologies MATech definition v2.0**

Medication Adherence Technologies (MATech) are evidence-based health technologies (i.e., devices, techniques, procedures/services, or systems) used in management of medication adherence by diverse stakeholders (i.e., patients, caregivers, healthcare professionals, *etc.*).

Particular elements used in this definition are described in more detail below.

• evidence-based encompasses the requirement of using available evidence for development of MATech as well as producing evidence that shows the contribution of the technology to medication adherence management;
• health technologies (i.e., devices, techniques, procedures/services or systems) emphasize the inclusion of all types of technologies aimed at managing medication adherence, irrespective of their mode of delivery or included technical elements/solutions;
• used in management of medication adherence by diverse stakeholders (i.e., patients, caregivers, healthcare professionals, *etc.*) encompasses the contribution of the technology to medication adherence management–either directly in patients’ self-management, or by supporting professionals in offering such services to patients through all types and phases of medication adherence.

### MAEI phase I definition

The Phase I definition of MAEI was a slight modification of the previous Phase 0 version (Kardas et al., 2022). To ensure consistency, the phrase “are broadly defined” was removed from its initial wording.

**Medication adherence enhancing intervention definition of phase I**

MAEIs are any formalised activities taking place within, or in association with the healthcare system, that in any way could positively affect medication adherence at the individual patient level.

### Reimbursement phase I definition

The Phase I definition of reimbursement was inspired by the ISPOR definition of reimbursement, which is the following: ‘Reimbursement relates to public or private insurers’ payment to providers for the delivery of healthcare products and services’ (Bingefors et al., 2003).

**Reimbursement definition of phase I**

Reimbursement relates to public or private insurers’ payment to providers for covering the costs of delivering MATechs and/or MAEIs.

### Best practice phase I definition

Discussion on the best practices was inspired by various available definitions of the best practices, i.e.,.

• “Best practices are health practices, methods, interventions, procedures or techniques based on high-quality evidence in order to obtain improved patient and health outcomes.” (Makic et al., 2013)
• “Best practice in healthcare are defined as the ‘best way’ to identify, collect, evaluate, disseminate, and implement information about as well as to monitor the outcomes of healthcare interventions for patients/population groups and defined indications or conditions.” (Perleth et al., 2001)
• According to WHO, “best practice” is commonly defined as “a technique or methodology that, through experience and research, has proven reliably to lead to a desired result.” It is also defined as “knowledge about what works in specific situations and contexts, without using inordinate resources to achieve the desired results, and which can be used to develop and implement solutions adapted to similar health problems in other situations and contexts.” (WHO, 2008).

However, it is noteworthy that the ‘best practice’ was not defined in Phase I.

#### Phase II

As many as 111 participants took part in the online stakeholder consultations, of which 75 participants came from 26 EU countries, and another 36 from non-EU countries. Approximately 2/3 of the survey participants were female (68.5%), mainly representing academia and healthcare sectors. Over 2/3 of the respondents had a PhD degree, and 64.0% were ENABLE members. For detailed characteristics of the respondents, see Table 2.

**TABLE 2:** Characteristics of the participants of the online stakeholder consultations.

	Values	Frequencies (%)
Gender (%)	Female	76 (68.5)
	Male	34 (30.6)
	Do not wish to answer	1 (0.9)
Age (%)	18–30	15 (13.5)
	31–40	25 (22.5)
	41–50	36 (32.4)
	51–60	18 (16.2)
	61–70	13 (11.7)
	70+	2 (1.8)
	Do not wish to answer	2 (1.8)
Country (%)	Albania	2 (1.8)
	Austria	2 (1.8)
	Belgium	2 (1.8)
	Bosnia and Herzegovina	2 (1.8)
	Bulgaria	3 (2.7)
	Croatia	3 (2.7)
	Cyprus	1 (0.9)
	Czech Republic	3 (2.7)
	Denmark	1 (0.9)
	Estonia	1 (0.9)
	Finland	1 (0.9)
	France	2 (1.8)
	Germany	3 (2.7)
	Hungary	4 (3.6)
	Iceland	1 (0.9)
	Ireland	3 (2.7)
	Israel	1 (0.9)
	Italy	4 (3.6)
	Lithuania	1 (0.9)
	Luxembourg	1 (0.9)
	Montenegro	1 (0.9)
	Netherlands	8 (7.2)
	North Macedonia	2 (1.8)
	Norway	2 (1.8)
	Poland	2 (1.8)
	Portugal	7 (6.3)
	Romania	4 (3.6)
	Serbia	1 (0.9)
	Slovakia	1 (0.9)
	Slovenia	2 (1.8)
	Spain	10 (9.0)
	Sweden	3 (2.7)
	Switzerland	4 (3.6)
	Turkey	3 (2.7)
	United Kingdom	8 (7.2)
	Other	12 (10.8)
Education (%)	Bachelor	1 (0.9)
	Doctorate (PhD)	76 (68.5)
	High school diploma	3 (2.7)
	Master	22 (19.8)
	Speciality Degree (healthcare)	9 (8.1)
Expertise (%)	Data Science/Statistics	4 (3.6)
	Economy/Management	1 (0.9)
	Medicine	20 (18.0)
	Nursing	9 (8.1)
	Pharmacy	63 (56.8)
	Psychology	4 (3.6)
	Sociology	1 (0.9)
	Other	9 (8.1)
ENABLE membership (%)	Yes	71 (64.0)
	No	40 (36.0)
TOTAL		111 (100.0)

Cumulative results of the stakeholder consultations are presented in Table 3. Of a note is that there were no significant differences in agreement and clarity among participants depending on their clinical or academic experience or ENABLE membership. Geographical differences could not be examined due to the limited number of participants from different countries. According to the low values of the Disagreement Index (range 0.12–0.38), none of the assessed terms or definitions was a subject of disagreement as to their clarity and content. Therefore, all the assessed items were retained in the target terminology.

**TABLE 3:** Median values and indicators of agreement for ratings of clarity and agreement regarding terms and definitions subject to the online stakeholder consultation.

Question	Outcome	Median	IPR	IPRAS	DI
MATech definition	Clarity	7.02	1.94	5.35	0.36
MATech definition	Agreement	7.01	1.77	5.49	0.32
MAEI term	Clarity	8.00	0.91	6.83	0.13
MAEI term	Agreement	7.97	1.23	6.30	0.20
MAEI definition	Clarity	7.04	1.98	5.30	0.37
MAEI definition	Agreement	6.98	1.72	4.95	0.35
Reimbursement definition	Clarity	7.57	1.24	6.07	0.20
Reimbursement definition	Agreement	6.99	2.05	5.32	0.38

Notes: IPR: Interpercentile Range 30–70, IPRAS: interpercentile range adjusted for symmetry, DI: Disagreement Index (ratio IPR/IPRAS; indicates disagreement if > 1).

#### MATech

In the online stakeholder consultations, MATech v2 definition received 39 comments and/or suggestions for clarification or modification. They referred to 4 main topics.

1. The term ‘evidence-based’. Some participants advised to exclude this term, while others commented on the difficulty to agree on which evidence would be recommended or sufficient for a technology to be considered a MATech. After careful consideration, it was decided that the term should remain unchanged as it aligns with the European Commission’s definition of best practice and the evidence base represents a specific domain in the framework defining the repository and the knowledge management system intended to accompany it.
2. The term ‘health technologies’ was suggested to impose too broad scope, and was suggested to be changed for procedures, services, *etc.*, as well as to be limited to an electronic or digital area only. Upon careful deliberation, it was determined that retaining the term in its current form is appropriate as it is widely accepted and easy to understand, also being inclusive enough to cover a broad range of contexts.
3. The term ‘medication adherence management’ was deemed inappropriate by some participants and some advised to replace it with terms specifying the promotion or enhancement of medication adherence. Upon detailed consideration, the decision was made to retain the term in its original form as it aligns with the ABC taxonomy and encompasses both measurement and intervention regarding medication adherence. To clarify this aspect, it was decided to include an explanatory text accompanying the definition.
4. The term ‘stakeholders’ was deemed sufficient and not requiring any examples in the definition sentence (rather in the explanatory text). ‘Different’ was suggested as a more appropriate adjective in English that ‘diverse’. This suggestion was considered for modification in v3 of the definition and explanatory text.

#### MAEI

The term MAEI received 17 comments in the online stakeholder consultations exercise, of which most suggested other options for ‘enhancing’, such as ‘supportive’, ‘promoting’, ‘enabling’ and ‘optimizing’. After careful consideration, it was determined that the term should remain unchanged due to its comprehensive meaning, encompassing both the promotion of high performance and the enhancement of current outcomes.

The definition of MAEI received 32 comments and/or suggestions for clarification or modification. They referred to 4 main topics.

1. The term ‘formalised’, which was suggested to be replaced by ‘structured’. The suggestion was accepted as it more accurately conveyed the original intentions of MAEI, which was to provide a framework that could be replicated consistently.
2. The term ‘within, or in association with the healthcare system’, which was questioned due to creating an unnecessary limitation, as some interventions could be provided without any association with a healthcare system. The decision was made to retain the term in its current form to prevent a loose interpretation of the relationship between the intervention and medication adherence, as broadening the definition would risk diluting its specificity (e.g., universal school education is certainly effective in developing the ability to understand the need for medication adherence among people, yet this is not an intervention targeting adherence directly, nor primarily).
3. The term ‘in any way’, which was suggested to be simply deleted. This suggestion was accepted to ensure the definition remains concise and clear.
4. The term ‘at individual patient level’, which was questioned due to the fact that potentially, adherence can be helped at a higher level, such as the healthcare facility or healthcare system level. After careful consideration, it was determined that the term should remain unchanged, as the entire concept of patient adherence, as defined by the ABC taxonomy, is centred around the individual patient, with his/her own characteristics, and promotes individualised approach to individual challenges. For example, general availability of more affordable drugs (e.g., generics) promotes adherence, yet it cannot be assumed to be an intervention designed and implemented for a particular combination of patient, condition, drug and external factors.

#### Reimbursement

In the online stakeholder consultations, definition of “reimbursement” in relation to MATechs/MAEIs received 34 comments referring to 6 main topics.

1. There exist multiple definitions of reimbursement for pharmaceuticals or medical devices. Therefore, respondents questioned the need for a separate reimbursement definition for MATechs/MAEIs. However, adherence technologies and interventions differ significantly from pharmaceuticals or medical devices, and hence, may require distinctive reimbursement considerations.
2. The term “public or private insurers” may not accurately reflect all the possible sources of reimbursement for MATechs/MAEIs. Therefore, this term was removed from the definition and instead a more inclusive wording was used that encompasses all relevant stakeholders who may finance these technologies and interventions.
3. Some respondents noted that payment pathways for adherence interventions may vary, and patients may also be eligible for reimbursement related to these interventions, not just providers. This feedback was taken into consideration and the definition was updated accordingly.
4. Suggestions were made to modify the term ‘covering the costs’ to reflect the extent of reimbursement, including whether it covers the entire cost or only a portion of it. Additionally, it was suggested to include cost elements in the definition. After careful consideration, we incorporated the extent of reimbursement (partly or entirely) in the definition. However, it was decided not to include cost elements, as it would overcomplicate the definition.
5. The term “delivering” was found to be unclear in the context of the definition. Therefore, it was decided to replace it with the term “providing” for more clarity.
6. Several respondents criticized the use of the terms “MATechs/MAEIs” in the definition of “reimbursement” due to a lack of understanding of the definitions of MATech and MAEI. Some suggested removing “MATechs” from the definition of “reimbursement”, arguing that technologies alone cannot improve medication adherence and therefore should not be the target of reimbursement. After defining MATech and MAEI, it was decided that both terms should be included in the definition.

### Best practice definition

In total, 71 respondents (81 comments collected) shared their opinions on the definition of “best practice” regarding MATech/MAEI, referring to 2 main merged topics.

1. Best practice should be outcome-oriented (42% of responses). Respondents believed that best practice represents MATechs/MAEIs which make most patients adherent to their medications, provide the best medication adherence results or improve medication adherence. Additional 14 comments expressed an opinion that MATech/MAEI should be cost-effective to be considered ‘best practice’.
2. Relation of best practice and evidence-based. Twenty-two respondents (33% of responses) related “best practice” to evidence-based. The use of general definition was suggested. As an option, the following was proposed: “Best practices are health practices, methods, interventions, procedures or techniques based on high-quality evidence in order to obtain improved patient and health outcomes”.

Respondents agreed that the best practice definition is related to both MATech/MAEI. Other comments and suggestions after content analyses were considered to be irrelevant to the definition of best practice.

#### Phase III

The iterative approach applied to the fine-tuning of the definitions allowed for designing the final set of definitions which constitute the ENABLE terminology.

In the first step, original verbatim transcripts of workshop #1 and #2 discussion recordings, onsite hand notes and chat notes allowed for identification of 55 comments, out of which as many as 61 comment items were extracted, and ascribed to each of the four definitions (Table 4). Online stakeholder consultations provided another 183 items, thus making the total number of comment items as high as 244. Through a meticulous content analysis, it was possible to cluster these items into 25 distinct groups. Out of these clusters, 6 pertained to the definition of “Best Practice”, 9 focused on defining MATech, while 5 referred to the definition of MAEI and another 5 were related to the definition of “Reimbursement”. However, only 5 of these clusters were assessed to be of high importance (“very useful”), and therefore, were subject of modifications of Phase II definitions, and approval at the forum of the cross-WG terminology working panel.

**TABLE 4:** Statistics of the process of final definition fine-tuning in Phase III.

Definition of	Items#			Clusters			
	Merged notes	Online stakeholder consultations	Total	Not relevant	To be considered	Very useful	Total
MATech	18	34	52	3	4	2	9
MAEI	25	44	69	2	1	2	5
Reimbursement	8	34	42	1	4	0	5
Best Practice$	10	71	81	1	4	1	6
TOTAL	61	183	244	7	13	5	25

Note: # items extracted from individual comments; one comment could be extracted from multiple items; $ No definition of ‘Best practice’ was available in Phase II; comments provided with regard to the dimensions that this definition was believed to cover.

#### MATech

In the case of the definition of MATech, two highly important suggestions concerned 1) exclusion of the list of technologies, and 2) exclusion of the list of stakeholders. Both of these options were accepted to simplify the definition, resulting in the final version of MATech definition v3.0.

#### MAEI

In the case of the definition of MAEI, two highly important suggestions concerned 1) changing ‘formalised’ into a more relevant term, e.g., ‘structured’, and 2) adding a reference to evidence to the definition. Both of these options were accepted because they conveyed the intended meaning of the definition more clearly. Additionally, the reference to evidence was consistent with the other elements of the final set of definitions.

#### Reimbursement

As regards the definition of ‘Reimbursement’, three crucial recommendations were made: 1) to exclude the list of stakeholders who may be responsible for paying the reimbursement, 2) to include patients as beneficiaries of the reimbursement, and 3) to incorporate the extent of reimbursement into the definition, regardless of whether it covers the entire cost or only its portion. All these recommendations were accepted, resulting in a simpler yet more comprehensive definition.

#### Best practice

In the case of the ‘Best Practice’ definition, only one highly important suggestion was found, namely, adding a reference to evidence to the definition. Similarly to the MAEI definition, this option was accepted because it conveyed the intended meaning of the definition more clearly.

In the WG1 workshop held on 29 March 2023 in Zagreb, Croatia, the members of the steering committee and the members of WG3 and WG4 groups discussed the issue of theories behind the term “best practice”. Afterwards, the following definition was suggested: “Best practice in adherence is evidence-based practice enhancing medication adherence”, where evidence-based practice is the integration of clinical expertise, patient values, and the best research evidence into the decision-making process for patient care (Sackett et al., 1996; Sackett et al., 2000). Consensus was reached through the online tool asking WG1 members to agree with the suggested definition. The 7-point Likert scale was used, where 1 indicated “strongly disagree” and 7 “strongly agree”. Points 5 to 7 were calculated as an agreement and consensus was reached at 80% (28 out of 35). According to the concluding suggestions from the panellists, the final definition was updated to: “Best practice in medication adherence is evidence-based practice enhancing medication adherence”.

Hence, the example of the best practice could be providing patients with feedback on their drug taking based on its electronic monitoring, due to clear evidence that such an approach is effective (Demonceau et al., 2013). On the other hand, relying solely on physicians’ assessments of their patients’ adherence is not a best practice as there is ample evidence that physicians fail to correctly identify which of their patients are non-adherent (Hines and Stone, 2016).

### Final set of definitions

The final set of definitions forms a cohesive taxonomy, as presented in Table 1, establishing an interconnected ecosystem. MATechs encompass various technologies that can be utilized in the context of MAEIs. A specific MAEI may incorporate one or multiple MATechs, while it is also conceivable to have MAEIs that do not rely on any MATechs (such as, e.g., medication regimen management-based interventions). Reimbursement represents a critical parameter for both MATechs and MAEIs, and best practice in medication adherence involves the practical application of MATechs and MAEIs in real-life settings. Therefore, within both scientific and clinical contexts, multiple terms from this taxonomy can be employed simultaneously.

## Discussion

Certainly, adherence itself is not the ultimate aim, but rather a means to achieve improved health outcomes. On the other hand, the link between the two is strong: the better the adherence, the greater the effectiveness of therapies. Therefore, given the current low levels of adherence, this factor becomes extremely important among the modifiable determinants of public health.

Unfortunately, despite half a century of adherence research, and a number of excellent publications devoted to the review of available approaches (Nieuwlaat et al., 2014; Mbuagbaw et al., 2015; Mistry et al., 2015; Morrissey et al., 2016), no consensus has yet been reached as to the terminology that should be used to describe medication adherence bettering activities. This scenario entails far-reaching consequences, ranging from hindering scientific research to negatively impacting the benchmarking of current interventions, and even inhibiting the adoption of best practices in healthcare policy. Consequently, available tools and methods are not promoted, and effective ways of supporting medication adherence are underused. This scenario is illustrated perfectly well by a recent survey conducted in 38 European countries and Israel, which identified 13 reimbursed MAEIs in nine countries only (Ágh et al., 2022).

The taxonomy proposed by ENABLE collaboration is a first set of cohesive terminology that attempts to cover this large gap. Being the result of an iterative process of fine tuning and co-design with stakeholders, it might be expected to lay conceptual foundations for more rigorous scientific research, and facilitate taking more objective and well-informed decisions in clinical practice and healthcare policy.

It is noteworthy that the final elements of the ENABLE taxonomy place great importance on evidence. This is not a coincidence. On the contrary, this is an approach similar to those adopted for general adherence terminology by the ABC taxonomy (Vrijens et al., 2012), and for reporting of the scientific studies by the EMERGE guidelines (De Geest et al., 2018). Therefore, these three guidance documents could be perceived as a cohesive ecosystem.

Moreover, we hope that the set of the definitions proposed by the ENABLE taxonomy is complete, and that there is no overlap between the individual terms. In particular, MATech stands for technological part of medication adherence bettering activity, whereas MAEI represents an entire intervention. Of course, most of the MAEIs use one or multiple MATechs. However, MATech may also be a stand-alone product, and finally, the same MATech might be applied in various MAEIs.

Of course, the proposed taxonomy has some limitations. Obvious one is the language used to express the terms and definitions. As it is currently only English, it may require validated translations into other languages ​​in the future. Moreover, the scope of the terminology is definitely reflecting European roots of the ENABLE collaboration, putting much attention to the dimension of reimbursement of adherence-enhancing actions. Indeed, in a short-term perspective, this taxonomy will be used by ENABLE in its own activities, such as the repository of MATechs, or further search of reimbursed MAEIs. For that reason, it prioritizes healthcare system-related perspective, putting much less attention to other (e.g., social) determinants of health. Specifically, it restricts the MAEI definition to those targeting individual patient level interventions. This approach excludes interventions at other levels, such as community-based initiatives. While such interventions can somehow impact adherence, assessing their effects accurately can be quite challenging. Finally, this first of its kind terminology needs extensive ‘real life testing’ regarding its usability and added value, that will come with further studies. Nonetheless, we firmly believe that it will prove useful to many stakeholders and, in a longer perspective, encourage further discussion on effective methods for promoting medication adherence.

## Data Availability

The datasets presented in this study can be found in online repositories. The names of the repository/repositories and accession number(s) can be found below: Zenodo - https://zenodo.org/record/8356621
